# Expression Profile Analysis of Circular RNAs in Leishmaniasis

**DOI:** 10.3390/tropicalmed7080176

**Published:** 2022-08-10

**Authors:** Zhongqiu Li, Wenbo Zeng, Yufeng Yang, Peijun Zhang, Zhengbing Zhou, Yuanyuan Li, Yunhai Guo, Yi Zhang

**Affiliations:** 1 National Institute of Parasitic Diseases, Chinese Center for Disease Control and Prevention (Chinese Center for Tropical Diseases Research), NHC Key Laboratory of Parasite and Vector Biology, WHO Collaborating Center for Tropical Diseases, National Center for International Research on Tropical Diseases, Shanghai 200025, China; 2 Rizhang Center for Disease Control and Prevention, Rizhao 276803, China; 3 Yangquan Center for Disease Control and Prevention, Yangquan 045000, China; 4 School of Global Health, Chinese Center for Tropical Diseases Research, Shanghai Jiao Tong University School of Medicine, Shanghai 200025, China

**Keywords:** leishmaniasis, circular RNA, neglected tropical diseases, zoonotic diseases

## Abstract

Leishmaniasis is a neglected tropical disease that seriously influences global public health. Among all the parasitic diseases, leishmaniasis is the third most common cause of morbidity after malaria and schistosomiasis. Circular RNAs (circRNAs) are a new type of noncoding RNAs that are involved in the regulation of biological and developmental processes. However, there is no published research on the function of circRNAs in leishmaniasis. This is the first study to explore the expression profiles of circRNAs in leishmaniasis. GO and KEGG analyses were performed to determine the potential function of the host genes of differentially expressed circRNAs. CircRNA–miRNA–mRNA (ceRNA) regulatory network analysis and protein–protein interaction (PPI) networks were analyzed by R software and the STRING database, respectively. A total of 4664 significant differentially expressed circRNAs were identified and compared to those in control groups; a total of 1931 were up-regulated and 2733 were down-regulated. The host genes of differentially expressed circRNAs were enriched in ubiquitin-mediated proteolysis, endocytosis, the MAPK signaling pathway, renal cell carcinoma, autophagy and the ErbB signaling pathway. Then, five hub genes (*BRCA1*, *CREBBP*, *EP300*, *PIK3R1*, and *CRK*) were identified. This study provides new evidence of the change of differentially expressed circRNAs and its potential function in leishmaniasis. These results may provide novel insights and evidence for the diagnosis and treatment of leishmaniasis.

## 1. Introduction

Leishmaniasis, a zoonotic disease, is one of the neglected tropical diseases that can seriously influence global public health [1]. There are three main types of leishmaniasis: visceral leishmaniasis, cutaneous leishmaniasis and mucocutaneous leishmaniasis. It is widely distributed around the world, present in 101 countries, and is transmitted through the bite of infected female sandflies, whose hosts are animals such as canids, rodents, marsupials, hyraxes, and human beings [2]. Leishmaniasis is an obligate intracellular pathogen that is mainly parasitic on host cells, and it is one of the parasitic diseases that can be dangerous to human health [3]. Among all the parasitic diseases, leishmaniasis is the third highest cause of morbidity after malaria and schistosomiasis in terms of disability adjusted life years (DALYs) [4]. However, it is the second most common cause of mortality after malaria. According to publication reports, there are 101 countries and approximately 350 million people living in leishmaniasis-endemic areas [5]. Most leishmaniasis patients live in impoverished areas, which makes prevention, diagnosis, and treatment very difficult [6]. Therefore, more than 2 million people are afflicted by leishmaniasis worldwide each year, resulting in an estimated 40,000 deaths [7]. This parasite also has a high recurrence rate; patients may relapse after 6–12 months despite receiving the appropriate treatment [8]. Moreover, if untreated, patients can develop multisystem disease or a secondary infection, potentially leading to death [9]. Detecting pathogens is the traditional way to diagnose leishmaniasis through the smear or culture tests of bone marrow aspirate [10]. However, bone marrow aspiration is not only painful, but its protozoa density is also extremely low, and it is easy to miss the diagnosis. In addition to this, visceral leishmaniasis has no specific clinical symptoms; it is easy to confuse it with other diseases, especially in endemic areas [11,12]. Therefore, it is also necessary to conduct epidemiological investigations. Furthermore, the occurrence of minimally symptomatic and completely asymptomatic and subclinical disease is considered an important aspect of the epidemiology of visceral leishmaniasis, which requires clinicians to determine more epidemiological characteristics of leishmaniasis [13]. Hence, it is important to research the mechanism of visceral leishmaniasis in order to find effective diagnostic biomarkers.

Circular RNAs (circRNAs) were first discovered in RNA viruses in 1976 [14]. With the development of high-throughput sequencing technology, thousands of circRNAs species have been detected, and this number is still increasing. Although circRNAs are a new type of the non-coding RNAs with covalent closed-loop structures, some circRNAs have protein-coding potential [15,16]. He et al. first discovered that circRNAs have protein-coding capacity in his research on the hepatitis D virus [17]. Published research shows that the post-translation products of circRNA can participate in multiple physiological processes in the human body, for example, preventing the linear translation product from being degraded by ubiquitin proteases [18]. Moreover, circRNAs can competitively bind with microRNA (miRNA) and act as a miRNA sponge, thereby affecting the regulation of miRNA on target genes [19]. Previous research has found that when circACVR2A is overexpressed, it can act as a sponge for miRNA-626 to regulate EYA4 gene expression through the enhancement of cell proliferation, migration, and invasion in bladder cancer cells [20]. Rong et al. found that has_circ_0002577 can act as an miR-197 sponge to regulate the proliferation and invasion of endometrial cancer cells [21]. CircRNAs are more resistant to exonuclease degradation than linear RNA. Therefore, circRNAs have greater stability [22]. Many studies have shown that they are related to various diseases, including cancer [23], cerebrovascular diseases [24], systemic lupus erythematosus [25] and so on. However, there is no published study on the function and molecular mechanisms of circRNAs in leishmaniasis.

Zoonotic diseases seriously influence human health through spillover effects from their impact on animals and the environment [26]. In order to reduce the harm of zoonotic diseases, this study, according to the different expressions of circRNAs in patients, finds biomarkers of leishmaniasis. Therefore, the differentially expressed circRNAs and miRNAs were screened by high-throughput sequencing. Furthermore, the functionals and pathways of host genes were analyzed by gene ontology (GO) and the Kyoto Encyclopedia of Genes and Genomes (KEGG). In addition, the correlation of all genes was performed though protein–protein interaction (PPI) and the competing endogenous RNAs (ceRNA) network. This study may provide assistance for leishmaniasis diagnosis and expand the horizons of targeted gene therapy.

## 2. Materials and Methods

### 2.1. Clinical Samples

According to data from the Infectious Disease Reporting Information Management System of the Chinese Center for Disease Control and Prevention, leishmaniasis is most prevalent in Yangquan, China. The sera of 3 leishmaniasis patients and 3 healthy persons (controls) were obtained from the Yangquan Center for Disease Control and Prevention.

### 2.2. RNA Extraction

Total RNA was extracted using TRIzol reagent (Thermo Fisher Scientific, Waltham, MA, USA). Additionally, the concentrations of RNA samples were measured, and purity was determined with an ultraviolet spectrophoto-meter (NanoDrop ND-1000, NanoDrop, Wilmington, DE, USA). RNA integrity and gDNA contamination were assessed by agarose gel electrophoresis.

### 2.3. Library Construction and RNA Sequence Analysis

The RNA libraries were constructed using RNA samples with the RNA Integrity Number (RIN) ≥ 8. Subsequently, the RNA libraries were controlled for quality by the BioAnalyzer 2100 system. The RNA-Seq libraries were performed in NovaSeq6000 (San Diego, CA, USA). RNA-seq data were analyzed using the Tuxedo protocol. The reads were aligned to GRCh38.p13 (http://asia.ensembl.org/Homo_sapiens/Info/Index (accessed on 12 March 2022)) by TopHat (version 2.1.1, Daehwan Kim and Steven Salzberg, MD, USA). Additionally, the transcript assembly and abundance were determined using Cufflinks (version 2.1.1, the lab of Cole Trapnell, Washington, DC, USA). circRNAs/miRNAs were analyzed as significantly different expressions with an absolute fold change ≥ 2 and with *p* < 0.05.

### 2.4. Screening od Differentially Expressed circRNAs and miRNAs

Differentially expressed circRNAs-/-miRNAs were determined and data were normalization using R software (v4.0.3, Hadley Wickham, IA, USA). A *t*-test was used to identify differentially expressed circRNAs and miRNAs with a significance level ≤ 0.05. The differentially expressed circRNAs and miRNAs thresholds were analyzed with a fold change ≥ 2. Moreover, the fold change and *p*-value were used to identify the top 10 up-and down-regulated differentially expressed circRNAs and miRNAs in leishmaniasis patients and local normal health persons. Volcano plots were visualized by ggplot2 in R software (v4.0.3).

### 2.5. Functional and Pathway Enrichment Analyses

Gene ontology (GO) analysis was used to highlight the biological processes (BP), molecular functions (MF), and cellular components (CC) of genes. Hence, the host genes of differentially expressed circRNAs were analyzed for their potential function. Kyoto Encyclopedia of Genes and Genomes (KEGG) pathway analyses were performed to find the functional attributes of the host genes using clusterProfiler (v4.0.2, Vince Carey, Boston, MA, USA); *p* < 0.05 was set as the statistically significant difference.

### 2.6. PPI Network Construction and Regulation ceRNA Network

The STRING database (https://string-db.org/ (accessed on 23 March 2022)), an online biological database, can display interactions of proteins and genes. Then, the potential relationship of hosts genes and proteins for differentially expressed circRNAs was determined. The PPI network (PPI score > 0.8) was used to accomplish this Cytoscape (version 3.6.1, NIGMS, Bethesda, USA). The interactions between circRNAs and miRNA were identified by TargetScan and miRanda databases. Subsequently, mRNAs were predicted through response elements-MiRNAs by miRTarBase (version 2, Hsien-Da Huang, HongKong, China), miRWalk(version 3, University of Heidelberg, Heidelberg, Germany), miRDB (Xiaowei Wang, Washington University School of Medicine in St. Louis, Washington, DC, USA) and TargetScan (version 7.1, Bartel laboratory, MA, USA) software. Finally, an endogenous competitive ceRNA (circRNAs–miRNA–mRNA) network was constructed using Cytoscape software, with a *p* value < 0.05.

## 3. Results

### 3.1. The Differential Expression of Serum circRNAs and miRNAs

**Figure 1:**
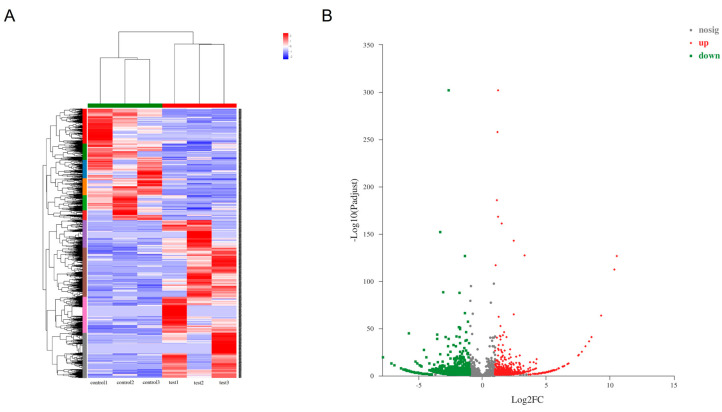
Differentially expressed circRNAs profiles. The heatmap (cluster analysis) was used to detect the significant differentially expressed circRNAs (**A**). Red represents high expression and blue represents low expression of circRNAs. Volcano plots were used to assess the circRNAs (**B**) expression variation. The values of X- and Y-axes in the scatter plot are averaged normalized values of each sample. Fold change ≥ 2, *p* < 0.05.

**Figure 2:**
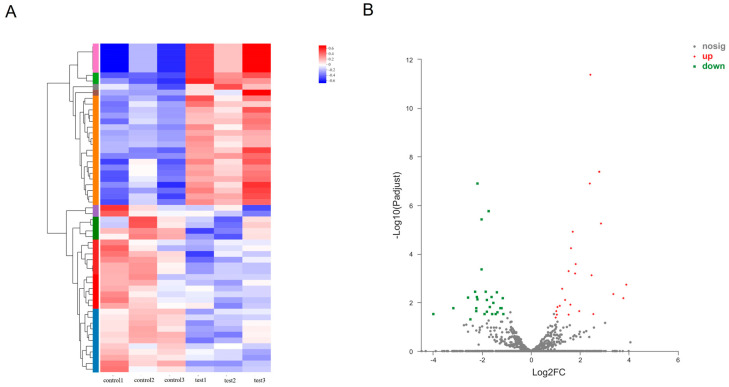
Differentially expressed miRNAs profiles. The heatmap (cluster analysis) was used to detect the significant differentially expressed miRNAs (**A**). Red represents high expression and blue represents low expression of miRNAs. Volcano plots were used to assess the miRNAs (**B**) expression variation. The values of X- and Y-axes in the scatter plot are averaged normalized values of each sample. Fold change ≥ 2, *p* < 0.05.

According to the statistical criteria of fold change ≥ 2 and *p* < 0.05, a total of 4664 significant differentially expressed circRNAs were identified compared with those in the control groups, of which 1931 were up-regulated and 2733 were down-regulated. Stratified cluster analysis showed that the circRNA expression patterns were distinguishable between leishmaniasis patients and healthy control group (Figure 1A), and volcano plots were used to show the significant differentially expressed circRNAs in the leishmaniasis patients’ group (Figure 1B). The top 10 up- (Table 1) and down-regulated (Table 2) circRNAs are listed in the tables in this paper. Under the same statistical screening conditions, there were 57 significant differentially expressed miRNAs, including 28 that were up-regulated and 29 that were down-regulated (Figure 2A,B). Similarly, the details of the top 10 up- (Table 3) and down-regulated (Table 4) differentially expressed miRNAs are listed.

### 3.2. Functional and Pathway Enrichment Analyses

**Figure 3:**
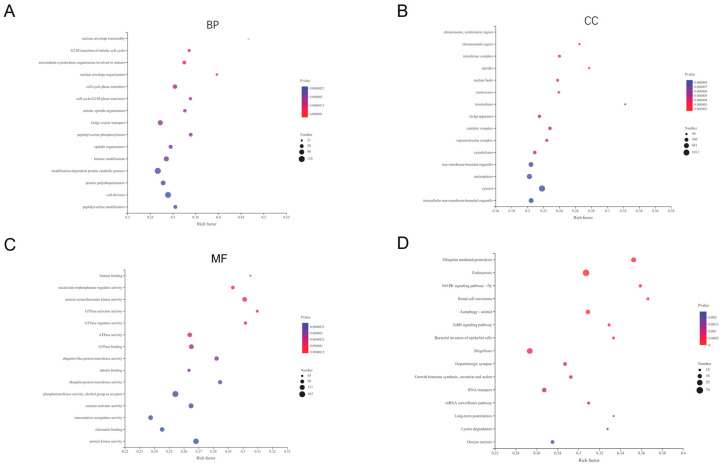
GO analysis and KEGG Pathway analysis. The target genes significantly enrich molecular function (MF), biological process (BP) and cellular component (CC) as shown in (**A**–**C**). The target genes significantly enrich signal pathways (**D**).

Briefly, GO analysis describes the host genes’ function and relationships between these. The enrichment results with regard to biological processes (BP) showed that nuclear envelope reassembly, G_2_/M transition of the mitotic cell cycle and microtubule cytoskeleton organization are involved in mitosis and other processes (Figure 3A). As for cellular components (CC), the host genes were enriched in chromosomes, the centromeric region, the chromosomal region and the transferase complex, as well as others (Figure 3B). Additionally, MF analysis showed that the host genes were enriched in histone binding, nucleoside-triphosphatase regulator activity and protein serine/threonine kinase activity, and others (Figure 3C). Furthermore, KEGG pathway enrichment analysis was enriched in some biological pathways, including ubiquitin-mediated proteolysis, endocytosis, MAPK signaling pathway, renal cell carcinoma, autophagy and the ErbB signaling pathway, and others (Figure 3D).

### 3.3. PPI Network Module Analysis of Host Genes

**Figure 4:**
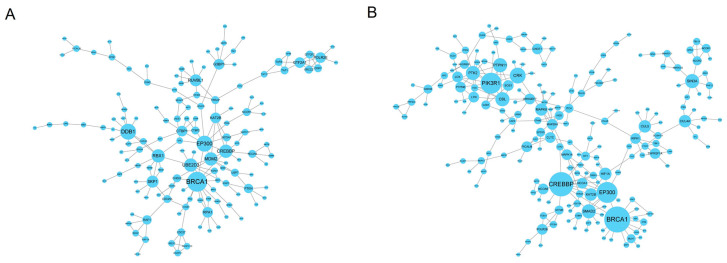
PPI network analysis of host genes. (**A**) The host genes of up-regulated circRNAs from the protein–protein interaction network. (**B**) The host genes of down-regulated circRNAs from the protein–protein interaction network.

A total of 142 nodes and 185 edges were found in down-regulated host genes with a PPI score ≥ 0.9 and experiments ≥ 0.6 (Figure 4A). In addition, 197 nodes and 254 edges were found in down-regulated host genes with a PPI score ≥ 0.9 and experiments ≥ 0.6 (Figure 4B). In total, the top five hub genes were calculated as hub genes using the plugin CytoHubba: *BRCA1*, *CREBBP*, *EP300*, *PIK3R1*, and *CRK*.

### 3.4. circRNA–miRNA–mRNA Network

**Figure 5:**
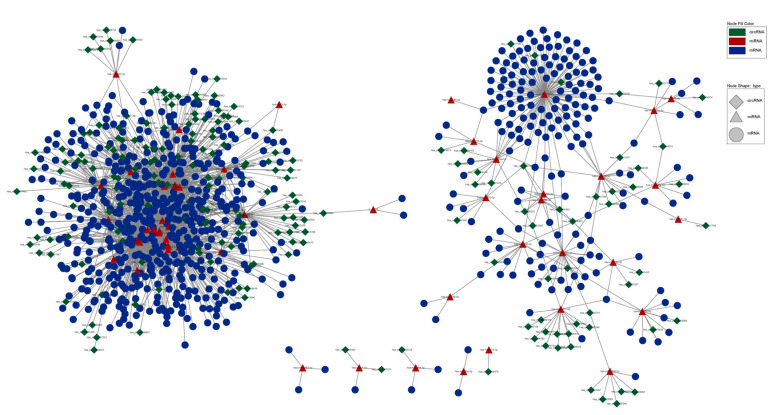
The circRNAs–miRNA–mRNA competing endogenous RNA network. Overall regulatory networks of circRNAs, miRNAs and mRNAs containing high-score interactions. Red triangles indicate up-regulated circRNAs, pink triangles indicate the down-regulated circRNAs, green circles represent miRNAs, blue rectangles indicate the mRNAs and a link between the nodes indicates the target relationship.

The ceRNA network was built to research the relationship among circRNAs, miRNAs and mRNAs. In total, 208 circRNAs, 52 miRNAs, 713 mRNAs, and 2034 edges had differentially expressed profiles, as shown in Figure 5.

## 4. Discussion

There is no published study on the function and molecular mechanisms of circRNAs in leishmaniasis. This is the first study to explore the different expressions of the circRNAs profile of leishmaniasis. In this study, a total of 4664 significant differentially expressed circRNAs were identified, which were compared with those in healthy persons through high-throughput sequencing, of which 1931 were up-regulated and 2733 were down-regulated. In recent years, many studies found that circRNAs have several special characteristics, including variety, structural stability, sequence conservation and specific expression that may play an important role in a variety of diseases [27], and participate in important biological processes, such as: neurogenesis [28], neuronal differentiation [29], and immune response [30,31]. Moreover, as a proto-oncogene or tumor-suppressor gene, circRNA is closely related to the occurrence of various tumors [32]. CircNUP210L (has_circ_0014359) influences on the miR-153/PI3K pathway to facilitate glioma cell proliferation [33]. As for lung cancer, Zhu et al. discovered specifically expressed circRNAs, 39 of which were up-regulated, and 20 were down-regulated [34]. They also found that has_circ_0013958 was up-regulated in the adenocarcinoma tissue and plasma of patients through further research; when knock down has_circ_0013958 is present, the proliferation and invasion ability of lung adenocarcinoma cells is promoted, and apoptosis is inhibited. However, circZFR plays the role of an oncogene by promoting the expression of CUL4B (cullin-4B) by sponging miR-101-3p [35]. In addition, Lai et al. found that has_circ_0047905, has_circ_0138960 and has_circRNA7690-15 are up-regulated in stomach cancer. The proliferation and invasion of gastric cancer cells are inhibited when knocking down the three circRNAs [36]. Furthermore, overexpressed circPSMC3 can inhibit the growth and proliferation of gastric cancer cells by targeting miR-654-3p and miR-296-5p, which affect the p21and PTEN signaling pathways [37]. Previous research found that the down-regulation of circTFF1 can inhibit the occurrence of breast cancer by sponging miR-326 and increasing the expression level of TFF1 [38]. Another study found that highly expressed circGFRA1 has a very important relationship with the prognosis of triple-negative breast cancer [39]. Qin et al. found that the expression of has_circ_0001649 is associated with tumor size and tumor emboli occurrence in hepatocellular carcinoma, which also suggests that has_circ_0001649 may serve as a potential new biomarker for hepatocellular carcinoma and play an important role in the occurrence and metastasis of hepatocellular carcinoma [40]. Furthermore, circUSP25 (hsa_circ_0001178) can induce hepatocellular carcinoma progression by regulating the miR-382/VEGFA axis [41]. However, circMALAT1 (hsa_circ_0002082) acts as a proto-oncogene or tumor-suppressor gene, and circMALAT1 can inhibit the translation of the tumor-suppressor gene PAX5 and also act as a sponge of miR-6887-3p, activate the JAK/STAT3 signaling pathway and promote the self-renewal of cancer stem cells [42]. The above research revealed that due to their characteristics, circRNAs can act as biomarkers in a variety of tumors. However, there is currently no research on the functions and mechanisms of circRNAs in leishmaniasis.

Therefore, this study discovered the functions of the host genes of significant differentially expressed circRNAs through GO and KEGG analyses. According to the results of the GO enrichment analysis, the host genes may play an important role in the occurrence of leishmaniasis. The results of KEGG pathway analysis showed that the top five significant signal pathways are enrich in ubiquitin-mediated proteolysis, endocytosis, the MAPK signaling pathway, renal cell carcinoma, autophagy and the ErbB signaling pathway. Among these five significant signaling pathways, three pathways have been shown to be associated with leishmaniasis. Previous research found that the endocytic pathway of *Leishmania* is achieved by clathrin, followed by the internalization of host hemoglobin (Hb) via a high-affinity receptor (HbR) [43]. Kumar et al. discovered that the endocytic pathway of *Leishmania* into host macrophages is through clathrin- and caveolin-mediated endocytosis [44]. In addition, *Leishmania* generally affects the CD40/MAPK pathway, and increases ERK1/2 and decreases IL-10 and IL-12 production [45]. Moreover, multiple studies demonstrate that enhanced LC3 labeling in vitro and in vivo is evidence that all *Leishmania* can induce autophagy [46,47,48]. Taken together, KEGG pathway analysis reveals the possible biological functions of these host genes of leishmaniasis.

In the present study, we also identify the five hub genes of those host genes of the PPI network, including: BRCA1, CREBBP, EP300, PIK3R1, and CRK. According to published literature reports, these 10 hub genes all play important roles in tumors or other diseases. However, only EP300 has been studied in leishmaniasis. Pragya et al. found that when *Leishmania* infected BMMFs, time-dependence is increased in EP300 binding to the Bcl2L12 promoter [49]. This suggests that the other four hub genes may also play important roles in leishmaniasis infection, which needs to be further studied.

## 5. Conclusions

This study is the first report of circRNA in leishmaniasis, and may provide novel insights and evidence for the diagnosis and treatment of leishmaniasis. However, these findings are preliminary, we will conduct further research on the corresponding circRNAs.

There are also several limitations in our study, the number of high-throughput sequencing samples in this study was relatively small and only met the requirements for publication. Moreover, the differentially expressed outer circular RNAs we screened require further validation in vivo and in vitro.

## Figures and Tables

**Table 1:** The top 10 up-regulated circRNAs in Leishmaniasis patients compared to healthy control group.

circRNA ID	logFC	*p* Value	Regulate	Significant
chr13: 30251931_30257867	10.54691	1.03 × 10^−130^	up	yes
chr 6: 31271073_31355592	10.32427	3.57 × 10^−116^	up	yes
chr 13: 30280063_30283791	9.30495	4.25 × 10^−67^	up	yes
chr 6: 29887955_29942626	8.557338	2.02 × 10^−44^	up	yes
chr 6: 29829418_29888742	8.551388	2.84 × 10^−44^	up	yes
chr13: 30621764_30647057	8.339884	2.45 × 10^−39^	up	yes
chr 1: 35850157_35851053	8.100131	2.25 × 10^−34^	up	yes
chr1: 244408712_244430099	7.772076	1.52 × 10^−28^	up	yes
chr 7: 77083887_77098951	7.563262	2.43 × 10^−25^	up	yes
chr 6: 3410188_3438555	7.515168	1.18 × 10^−24^	up	yes

**Table 2:** The top 10 down-regulated circRNAs in leishmaniasis patients compared to healthy control group.

circRNA ID	logFC	*p* Value	Regulate	Significant
chr 6: 32521905_32581838	−7.77687	1.49 × 10^−22^	down	yes
chr 12: 2866393_2872244	−7.14883	7.86 × 10^−16^	down	yes
chr17: 16034765_16040494	−7.14883	7.86 × 10^−16^	down	yes
chr 1: 11016844_11020599	−6.89104	1.11 × 10^−13^	down	yes
chr11: 20407911_20426865	−6.36747	3.68 × 10^−10^	down	yes
chr17: 63666940_63685578	−6.36747	3.68 × 10^−10^	down	yes
chr 1: 23971573_23972012	−6.17483	4.15 × 10^−9^	down	yes
chr2: 171435083_171458075	−6.12236	7.68 × 10^−9^	down	yes
chr1: 235812971_235833667	−6.06791	1.43 × 10^−8^	down	yes
chr12: 102269600_102315490	−6.06791	1.43 × 10^−8^	down	yes

**Table 3:** The top 10 up-regulated miRNAs in leishmaniasis patients compared to healthy control group.

miRNA ID	logFC	*p* Value	Regulate	Significant
hsa-miR-483-3p	3.906015	6.71 × 10^−5^	up	yes
hsa-let-7b-3p	3.784429	0.000352	up	yes
hsa-miR-486-3p	3.369297	0.000199	up	yes
hsa-miR-16-2-3p	2.870342	1.19 × 10^−7^	up	yes
hsa-miR-25-5p	2.467263	2.58 × 10^−5^	up	yes
hsa-let-7d-3p	2.430329	7.36 × 10^−15^	up	yes
hsa-miR-5100	2.400199	1.86 × 10^−9^	up	yes
hsa-miR-6877-5p	1.977799	0.001816	up	yes
hsa-miR-1260b	1.82933	6.81 × 10^−6^	up	yes
hsa-miR-877-5p	1.79051	2.06 × 10^−5^	up	yes

**Table 4:** The top 10 down-regulated miRNAs in leishmaniasis patients compared to healthy control group.

miRNA ID	logFC	*p* Value	Regulate	Significant
hsa-miR-4482-5p	−4.00131	0.002571	down	yes
hsa-miR-411-5p	−3.16776	0.001176	down	yes
hsa-miR-487b-3p	−2.57252	0.000307	down	yes
hsa-miR-381-3p	−2.47089	0.005327	down	yes
hsa-miR-654-3p	−2.29163	0.000147	down	yes
hsa-miR-2355-3p	−2.24553	0.00178	down	yes
hsa-miR-382-5p	−2.24167	0.001283	down	yes
hsa-miR-494-3p	−2.21251	0.000269	down	yes
hsa-miR-1-3p	−2.18407	0.000411	down	yes
hsa-miR-146a-5p	−2.1812	1.68 × 10^−9^	down	yes

## Data Availability

The RNA sequencing data has been uploaded to the SRA database (https://www.ncbi.nlm.nih.gov/sra/?term= (accessed on 26 April 2022)). With accession number: PRJNA824834 and PRJNA827265.
